# Microwave-assisted one-step synthesis of polyacrylamide/NiO nanocomposite for biomedical applications†

**DOI:** 10.1039/d5ra02496j

**Published:** 2025-06-05

**Authors:** Muhammed Yusuf Miah, Sukanta Halder, Shassatha Paul Saikat, Sanchita Dewanjee, Md. Ashaduzzaman, Shukanta Bhowmik

**Affiliations:** a Department of Applied Chemistry and Chemical Engineering, Noakhali Science and Technology University Noakhali-3814 Bangladesh shukantabhowmik1@nstu.edu.bd; b Department of Applied Chemistry and Chemical Engineering, University of Dhaka Dhaka 1000 Bangladesh azaman01@du.ac.bd

## Abstract

The study presents the successful one-step synthesis of a polyacrylamide/NiO nanocomposite (PAM/NiO NC) through the simultaneous formation of NiO nanoparticles (NiO NPs) and the polymerization of acrylamide (AM), which was carried out using the respective metal salt and AM monomer in ethylene glycol (EG) under microwave-assisted heating. The formation of the PAM/NiO NC was confirmed through UV-vis absorption spectroscopy, Fourier Transform Infrared (FTIR) spectroscopy, X-ray diffraction (XRD), and scanning electron microscopy (SEM) analyses. XRD data were used to calculate various crystallographic parameters, including crystallite size (*via* the Scherrer equation and other models), dislocation density, crystallinity, residual stress, and microstrain, and the analysis showed that the generated PAM/NiO NC has crystallite sizes ranging from 1 to 100 nm, within the accepted range. The crystallinity analysis revealed that the synthesized NC is semi-crystalline, with an average particle size of 26.33 nm, as determined by the Scherrer equation. As synthesized, NC exhibited excellent antibacterial activity against both Gram-negative (*Klebsiella* spp.*SK4*, *E. coli RN89*) and Gram-positive (*Streptococcus aureus-8a*) bacterial strains with the highest bactericidal capability against *Klebsiella* spp*.SK4* irrespective of concentration. *In vitro* cytotoxicity study revealed the strongest toxicity against HELA cell lines by PAM/NiO NC and no toxicity towards BHK-21 cell lines indicating PAM/NiO NC potential for biomedical applications.

## Abstract 2

Microwave-assisted one-step synthesis of PAM/NiO NC has been demonstrated as a fast, uniform, energy-efficient, and environment-friendly approach.

## Graphical abstract

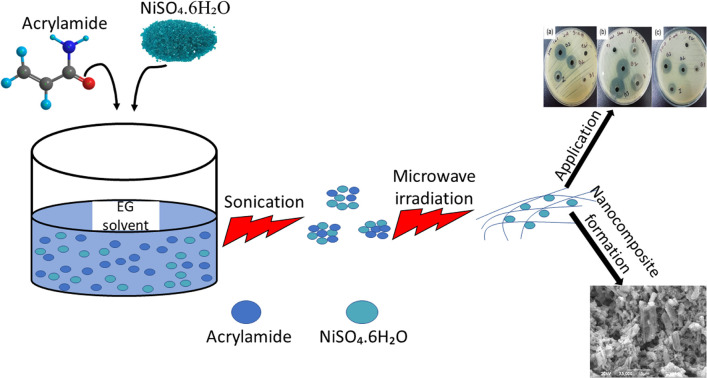

## Introduction

The incorporation of metal nanoparticles (NPs) into a polymer matrix results in the formation of polymer-metal nanocomposites, which have attracted boundless scientific interest and attention over the last few years due to their remarkable cytotoxic, antimicrobial and photocatalytic performance in the field of nanoscience, nanotechnology, biotechnology and medicine.^1–5^ Polymer/metal nanocomposites incorporate metal nanoparticles stably and additionally enhance other characteristics of the polymer.^6^ Combining the nanoscale dimensions of metal NPs with the characteristics of polymeric materials results in hybrid materials that exhibit unique and diverse mechanical,^7^ thermal, and optical properties.^8,9^

Despite the extensive application potential of polymer/metal nanocomposites, challenges persist in their synthesis. Conventionally, organic monomer polymerisation and metal nanoparticles synthesis have been conducted independently before being combined through various physical, chemical, or biomimetic approaches. This often results in inhomogeneous dispersion, severe nanoparticle aggregation, and inconsistent composite properties. Additionally, some methods involve the *in situ* synthesis of nanoparticles in the presence of a polymer or with its subsequent addition, where nanocomposites are formed by dispersing nanoparticles within a polymer solution followed by solvent evaporation or co-precipitation.^10–12^ These methods typically require high temperatures or pressures, making the preparation process complex, challenging, and costly. A one-step process for both metal NPs synthesis and monomer polymerization can facilitate the uniform dispersion of metal NPs with a narrow size distribution within the polymer matrix. Limited studies have explored the concurrent polymerization of monomers and the formation of metal NPs utilizing γ-ray irradiation,^13–16^ UV lamp,^17,18^ microwave equipment,^19^ additives, protective gas, or long preparation time.^20^ Among all these, the need for a cost-effective and facile method welcomed the new era of microwave heating in the synthetic chemical industry. Utilization of microwave heating for polymerization and formation of NPs is a very promising approach for its selectivity, high volumetric heating, and reduced reaction time.^19^ Nevertheless, despite the aforementioned advantages, the use of microwave chemistry for the one-step simultaneous synthesis of polymer-metal NC remains largely unexplored.

In this study, polyacrylamide/NiO nanocomposite (PAM/NiO NC) was synthesized using a one-step microwave heating process, resulting in a uniform distribution of metal NPs within the polymer matrix. Ethylene glycol (EG) acts as a solvent in the one-step microwave-assisted synthesis, dissolving both nickel sulfate hexahydrate (NiSO_4_·6H_2_O) and acrylamide (AM) monomers to create a homogeneous reaction medium.^21^ PAM, a well-known synthetic polymer with widespread applications in industries such as food, cosmetics, and water treatment, was selected as the polymer matrix due to its versatility. Furthermore, PAM's properties can be improved by integrating various chemical elements or copolymers. This makes PAM an ideal candidate for the development of NC, which has become an area of growing research interest,^19,22–24^ PAM/NiO NC has never been studied before to the best of our knowledge. Among transition metal oxide (TMO) NPs, NiO is a particularly essential material, characterized by a cubic lattice structure and distinct optical, thermal, electrical, chemical, and physical properties.^25^ Nickel oxide nanoparticles (NiO NPs) exhibit diverse applications across various fields, including their use in battery electrodes,^26^ photoelectronic devices,^27^ ion storage materials,^28^ gas sensors,^29^ magnetic and thermoelectric materials,^30^ catalysts,^31^ fuel cells,^32^ dye-sensitized photocathodes,^33^ electrochromic films,^34^ as well as in anticancer and cytotoxic activities.^35^ Additionally, NiO NPs are utilized in non-enzymatic glucose sensors.^36,37^

This research utilized a one-step method to concurrently synthesize NiO NPs and PAM, with EG serving as both the reducing agent and solvent, eliminating the need for additional reductants. The formation of semi-crystalline NC was confirmed by UV-vis, FTIR, SEM, and XRD analysis. The semicrystalline nature of nanocomposite plays a vital role in biomedical applications by providing a balance between mechanical strength and flexibility. This physical property is important for maintaining structural integrity, while functionally, it enhances tissue compatibility and implant stability.^38–40^ Furthermore, neither an initiator for acrylamide polymerization nor a surfactant for NiO NPs stabilization was required. This approach circumvented the need for complex post-synthesis purification steps, enabling a rapid, straightforward, and cost-effective synthesis of the PAM/NiO NC. The primary objective of this research was the successful fabrication of PAM/NiO NC and the exploration of its potential applications.

## Experimental section

### Materials

The chemicals utilized in the experiments were of analytical grade and were used without any further purification. Acrylamide (AM, min. assay 99%) was purchased from Qualikems Fine Chem. Pvt. Ltd. (Vadodara, India). Nickel sulphate hexahydrate (NiSO_4_·6H_2_O, assay 96%) and ethylene glycol (EG) were purchased from Merck-KgaA (Darmstadt, Germany). The sonicator used was Power Sonic 505, 5.7 L, Hwashin Technology Co., (Seoul, Korea), the hot plate was LMS-003, Daihan Labtech Co. Ltd. (Seoul, Korea), the microwave heater was made by Miyako, MD-80D20ATL-DJ, 800 MW, Japan and the oven used was made by Han Yang Scientific Equipment Co. Ltd. (Seoul, Korea).

### PAM/NiO NC synthesis

**Fig. 1:**
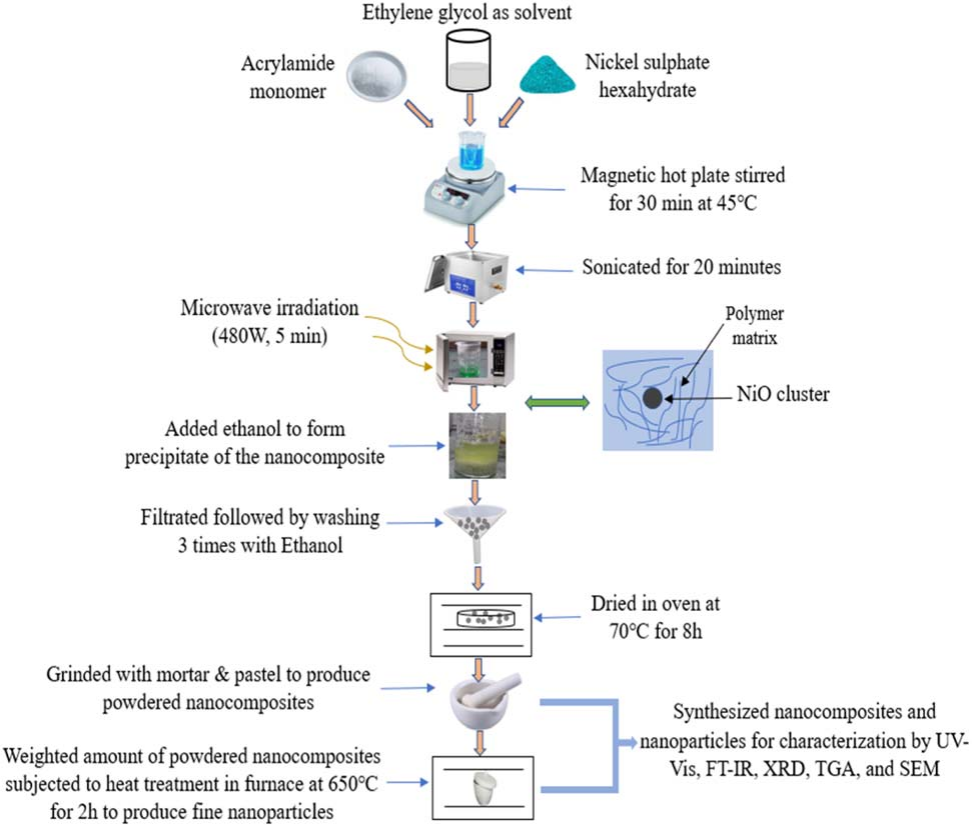
Synthesis route of PAM/NiO NC by microwave technology.

For the preparation of PAM/NiO NC, 6.39 mg of AM (3 M) and 2.13 mg of NiSO_4_·6H_2_O were dissolved in 20 mL EG. Later, the solution was sonicated in a water bath of a sonicator for 20 min to facilitate the mixing of components after being stirred in a hot plate magnetic stirrer for 30 min at room temperature. The solution was then rapidly microwave-heated (operated at 480 W) for 5 min that produced a more concentrated solution. The formation of polymer and nanoparticle was carried out *in situ*. The rapid microwave heating causes the thermal decomposition of NiSO_4_·6H_2_O and produced NiO NPs. The concentrated solution was filtered using ultra-filter paper, washed three times with 50 mL of ethanol, dried in an oven at 70 °C for 8 h, and subsequently ground into a powder using a mortar and pestle. Fig. 1 represents the synthesis route of PAM/NiO NC.

### Characterization

The powdered PAM/NiO NC was characterized to examine structural, optical, and nanostructural characteristics. Spectra of optical absorption were obtained using a UV-2100PC UV-vis absorption spectrometer (Human Lab Instruments, Gyeonggi, Korea). The bonding interactions and chemical composition of the synthesized polymer-metal oxide NC were evaluated *via* FTIR spectroscopy using an FTIR 8400S spectrophotometer (Shimadzu Corporation, Japan), with measurements taken in the wavenumber range of 4000–400 cm^−1^, a resolution of 2 cm^−1^, and 30 scans. The structural characteristics of PAM/NiO NC and pure PAM were evaluated using a Rigaku Ultima IV X-ray diffractometer (Tokyo, Japan) with Cu-Kα radiation (*λ* = 1.5406 Å). X-ray diffraction (XRD) measurements were conducted over a 2*θ* range of 10° to 70°, under 40 kV and 40 mA, with a scan speed of 3° min^−1^. Surface morphology, purity, and elemental compositions were analyzed using a JEOL JSM-6490LA scanning electron microscopy (SEM) (Tokyo, Japan) and a Shimadzu TGA-50 thermo-gravimetric analyzer (TGA) (Kyoto, Japan). Thermograms were recorded under a nitrogen atmosphere in an alumina pan (10 °C min^−1^, 25 °C to 800 °C).

### Antimicrobial activity evaluation

The evaluation of antimicrobial activity is an important aspect of assessing nanomaterials for biomedical and environmental applications.^41^ By testing the inhibitory effects of the PAM/NiO nanocomposites against selected microbial strains, the study aims to determine their effectiveness in preventing microbial growth and addressing concerns related to antimicrobial resistance. The antimicrobial properties of PAM/NiO NC were assessed against both Gram-positive (*Staphylococcus aureus-8a*) and Gram-negative (*Klebsiella* spp.*SK4, and Escherichia coli RN89*) bacterial strains. Antimicrobial susceptibility was determined for three bacterial isolates using the Kirby–Bauer disk diffusion method, as described in this study.^36,42^ Three different concentrations were employed: 5 g L^−1^, 20 g L^−1^, and 30 g L^−1^, and it contains 25% w/w NiO concentrations of 1.25 g L^−1^, 5 g L^−1^, and 7.5 g L^−1^. All samples were prepared by dissolving the required concentrations in distilled water, followed by sonication for 15 minutes. The optical density (OD) was adjusted to a range of 0.1–0.5 using a spectrophotometer. *Escherichia coli* DH5α was employed as the reference strain, while ampicillin was used as the control in the microbial assay. Four well-isolated colonies exhibiting similar morphology were selected from an agar culture plate, and a sterile loop was utilized to transfer the upper portion of each colony into a tube comprising 5 mL of Mueller–Hinton broth. The bacterial suspension was incubated at 37 °C. A sterile cotton swab was used to spread a uniform layer of bacteria, and wells were created using a serial cork borer. Next, 40 μL of the diluted samples was added to each well, and the plate was incubated at 37 °C for 24 hours.

### Cytotoxicity study

Cytotoxicity analysis is a critical step in evaluating the biocompatibility of nanomaterials intended for biomedical applications. It provides insight into the potential toxic effects of the material on living cells and helps determine its suitability for *in vitro* and *in vivo* use.^43^ For the cytotoxicity testing, various materials such as 96-well plates, 15-mL tubes, pipette tips, gloves, culture flasks, cell culture media, antibiotics (penicillin and streptomycin), gentamycin, serological pipettes, and trypsin were employed. The experiments were conducted at the Centre for Advanced Research in Sciences, University of Dhaka, Bangladesh, utilizing their commercial services. Three cell lines were used: VERO cells (kidney epithelial cells from the African green monkey), HeLa cells (human cervical carcinoma cells), and BHK-21 cells (baby hamster kidney fibroblast cells), which were purchased from American Type Culture Collection (ATCC, USA), and all of which were maintained in Dulbecco's Modified Eagle's Medium (DMEM) containing 1% penicillin-streptomycin (1 : 1), 0.2% gentamycin, and 10% fetal bovine serum (FBS). To test the cytotoxicity of the synthesized PAM/NiO nanocomposite (NC), VERO cells (1.5 × 10^4^/100 μL), HeLa cells (2.0 × 10^4^/100 μL), and BHK-21 cells (1.5 × 10^4^/100 μL) were seeded in a 96-well plate and incubated at 37 °C in a 5% CO_2_ environment. After 24 hours, 25 μL of the autoclaved sample was introduced into each well. Following 48 hours of incubation, any undissolved samples were discarded and replaced with fresh media, after which the cytotoxicity was assessed under an inverted light microscope (Optika, Italy).

### Photocatalytic performance evaluation

Photocatalytic activity evaluation is essential to determine the efficiency of nanomaterials in degrading organic pollutants under light irradiation and serves as a measure of the material’s potential in environmental remediation applications.^44,45^ To assess the photocatalytic performance of PAM/NiO NC under natural sunlight, Remazol Yellow RR dye was selected as a model pollutant. A 40 ppm stock solution of Remazol Yellow RR was prepared in deionized water, and the initial dye concentration was confirmed using a UV-Vis spectrophotometer at its maximum absorption wavelength (*λ*_max_) of 420 nm. The pH of the dye solution was adjusted to 6.5 using dilute HCl as required. For the photocatalytic experiments, 100 mL of the dye solution was placed in a 100 mL beaker, and PAM/NiO NC was added at a concentration of 0.2 g L^−1^. The mixture was stirred in the dark for 30 minutes to achieve adsorption–desorption equilibrium before exposure to sunlight. The beaker was then exposed to natural sunlight from 6:40 AM to 5:20 PM, during which the total solar radiation was approximately 5335 Wh m^−2^ with an average irradiance of around 370 W m^−2^. At two-hour intervals, 5 mL samples were withdrawn, filtered to remove the photocatalyst, and analyzed for absorbance at 420 nm using a UV-vis spectrophotometer. After irradiation, the residual dye concentration was assessed by measuring the absorbance at the dye's peak wavelength, and the percent degradation of the dye solution was calculated using eqn (2).^46^1
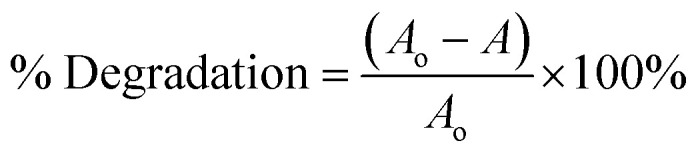
In this context, *A*_0_ and *A* denote the dye solution absorbance at 420 nm pre- and post-irradiation, respectively.

## Results and discussion

### Characterization

#### UV-visible spectroscopy

**Fig. 2:**
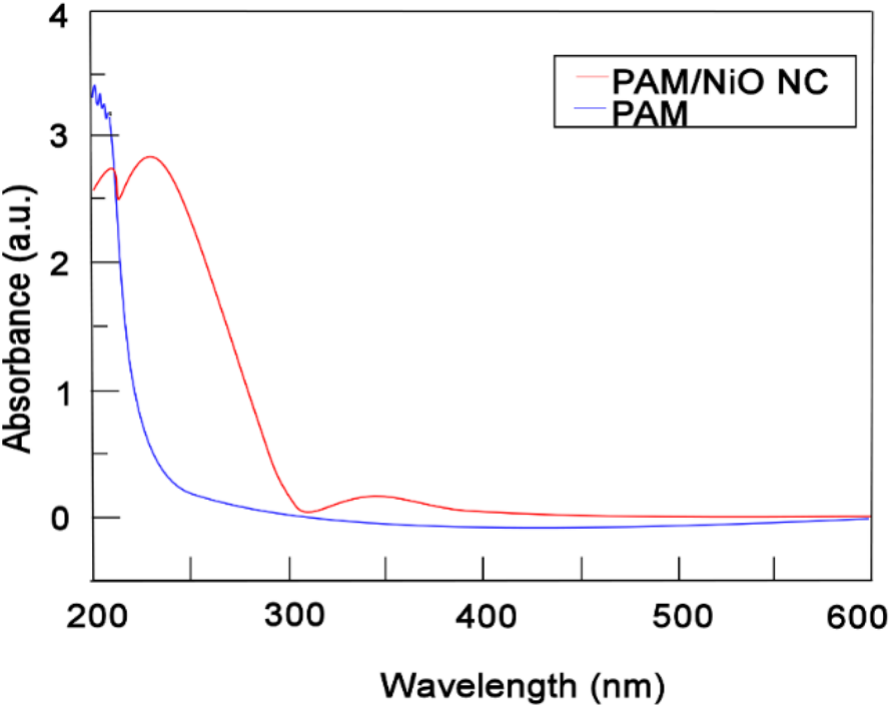
UV-vis spectra of PAM/NiO NCs & PAM.

The UV-visible spectrums of synthesised polyacrylamide and PAM/NiO NC are shown in Fig. 2, and S1 in the ESI data† represents the NiO NPs spectrum result. The absorption spectrum of PAM/NiO NC depends on several parameters such as method of fabrication, temperature and size of particles.

UV absorbance values of polyacrylamide generally lay between the wavelength ranges of 190–240 nm.^47^ Here, the spectrum of pure PAM took a curvature trend with a maximum absorbance at 190 nm, which confirms the successful generation of PAM polymer from AM monomer in the study.

Interestingly, in PAM/NiO NC, two broad humps are observed at the wavelength range of 220–260 nm and 320–380 nm, respectively. Absorption peaks for NiO NPs were found at various wavelengths ranging from 268–341 nm in reports of others.^36,42,48^ Thus, the initial absorption peak primarily results from the interaction between NiO NPs and PAM. This can be attributed to the variations in the absorption spectra of the individual components. The later one is of NiO NPs, as PAM shows almost no absorbance beyond 240 nm.^47^ That confirms the successful formation of PAM-NiO NC through this aforementioned method.

#### FT-IR analysis

**Fig. 3:**
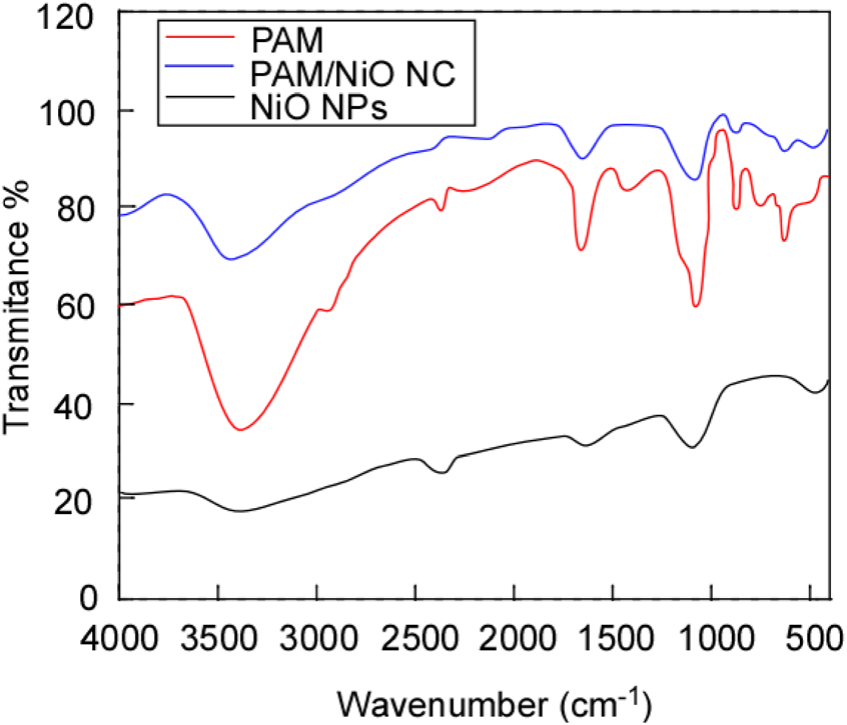
FT-IR spectra of PAM/NiO NC & polyacrylamide.

To confirm the incorporation of PAM and NiO in the NC, the FTIR spectra of both pure PAM and PAM/NiO NC, presented in Fig. 3, were examined. The peaks at *A* (578 cm^−1^) and *B* (726 cm^−1^) in the PAM/NiO NC were attributed to the vibration of the Ni–O bond,^36^ and the identification of a metal oxide linkage, respectively.^49^

The FTIR spectrum of the NC shows slight shifts in the characteristic peaks of PAM, which can be attributed to the bonding interaction between NiO and the polymer. The spectra of pure PAM and PAM/NiO NC exhibit similarity and correspond to the standard infrared spectrum of PAM.^19^ The peaks at 1085 and 1087 cm^−1^ in spectrum are ascribed to the stretching vibration of C–O, caused by the adsorption of atmospheric CO_2_ or ethanol. However, the intensity of this band is diminished in the NC, indicating that the ultrafine particles tend to physically adsorb H_2_O and CO_2_.^50^ The absorption band observed at *D* (∼1660 cm^−1^) assigned to the C=O stretching vibration in the –CONH_2_ group. Band *E* (2929–2958 cm^−1^) were attributed to –CH_2_– stretching mode^51^ and the asymmetric and symmetric N–H stretching vibrations were observed at *F* (3387–3398 cm^−1^) in PAM and PAM/NiO NC, respectively.^19^

#### X-ray diffraction (XRD) analysis

**Fig. 4:**
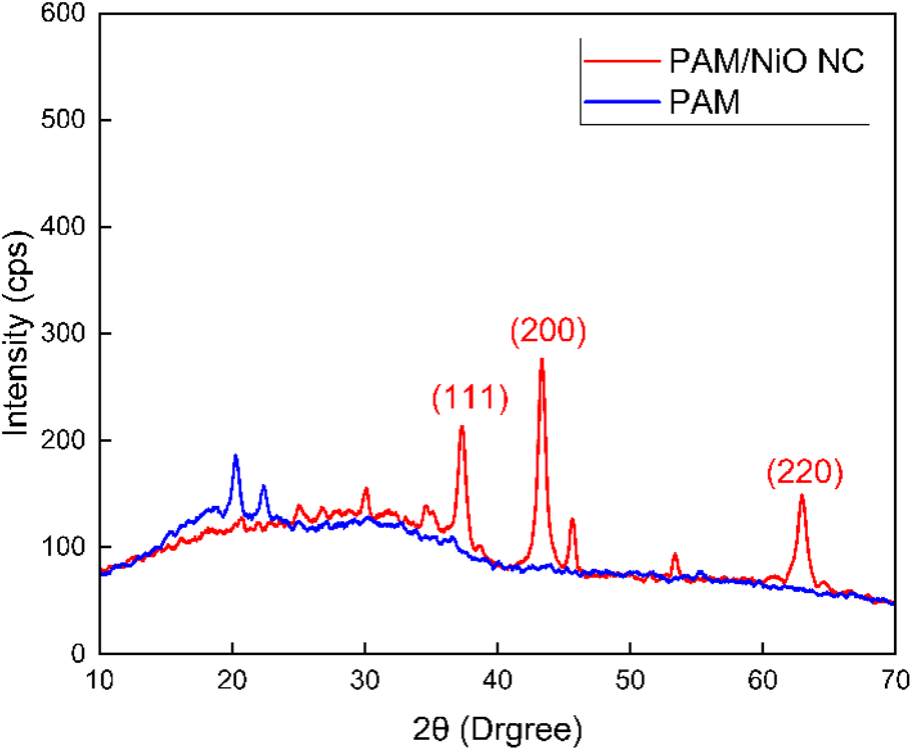
X-ray diffractogram of the synthesized PAM and PAM/NiO NC.

XRD patterns of pure PAM and the synthesized PAM/NiO NC through microwave heating are represented in Fig. 4, and the pattern of NiO NPs are given in Fig. S2 as ESI data.† Broad peaks centred at 2*θ* = 22.29° in both XRD patterns of PAM and PAM/NiO NC attributed to the PAM polymer phase,^19^ confirming that the polymerisation of acrylamide monomer happened in given experimental conditions. All other prominent diffraction peaks of PAM/NiO NC at 2*θ* values of 37.24°, 43.36°, and 62.86° can be clearly assigned to the (111), (200), and (220) crystal planes of bulk NiO, respectively.^48,52^ All of these reflections are consistent with the face-centered cubic (fcc) NiO phase, in perfect alignment with the standard data (JCPDS card no. 47-1049),^50^ confirming the successful synthesis of NiO NPs using EG as a reducing agent. As a result, the formation of the PAM/NiO NC has been confirmed, and the clarity and intensity of the peaks reflect the high crystallinity of the synthesized NPs.

Diffraction peaks corresponding to Ni_2_O_3_ (2*θ* = 32.2°, JCPDS card no. 14-0481)^53^ and nano-sized Ni (2*θ* = 48.77°)^54^ were observed in the Fig. 3. There was no other significant peak in the XRD pattern, which confirmed the purity of the nanocomposite sample. The average particle size of PAM/NiO NC was calculated at 26.33 nm using the Scherrer formula of eqn (2) from measured values of the XRD pattern.2
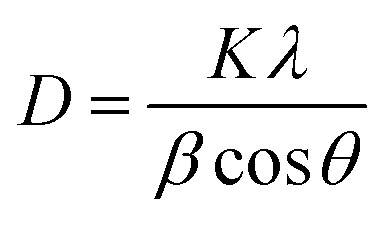
In the given equation, *K* represents the Scherrer constant, *λ* (lambda) denotes the wavelength of the incident light utilized in diffraction, *β* (beta) corresponds to the full width at half maximum (FWHM) of the sharp diffraction peaks, and *θ* (theta) signifies the diffraction angle. The Scherrer constant (*K*) accounts for the influence of particle shape and is conventionally assigned a value of 0.9.

The degree of crystallinity, which quantifies the proportion of the crystalline phase within the sample volume, can be expressed using the following eqn (3).

Crystallinity degree,3
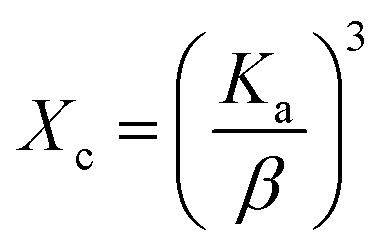


Saikiran *et al.* stated that dislocation density, which is associated with crystallite size, is a parameter that quantifies the number of dislocation lines per unit surface area.^55^ The following eqn (4) used the size of the crystallites to determine the dislocation density. Dislocation density,4
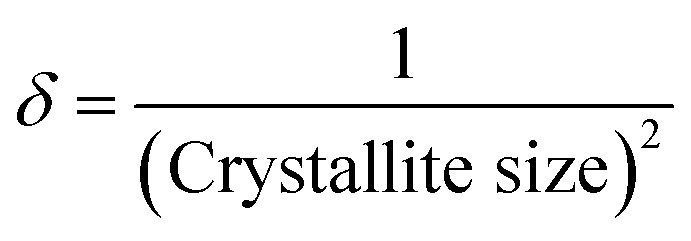


Microstrain, also referred to as local strain, refers to the changes in the lattice parameters of crystalline materials. It can be determined using eqn (5). Microstrain, 5
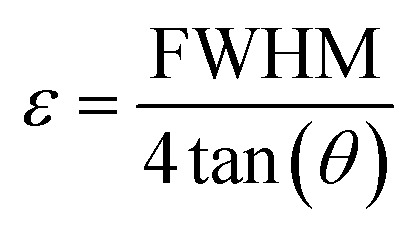
In this context, *D* represents the crystallite size in nanometers, *β* (beta) denotes the full width at half maximum (FWHM) in radians, *X*_c_ corresponds to the degree of crystallinity, *θ* refers to the diffraction angle in degrees, and *δ* signifies the dislocation density. Furthermore, *ε* represents the microstrain, the shape factor (an arbitrary constant) or Scherrer's constant, with *K* assigned a value of 0.94. The peak height of the corresponding plane is denoted as *H*, while *K*_a_ is given as 0.24.

Table 1 represents the different crystallographic parameters of the produced PAM/NiO NC by Scherrer equation. The specific values of *β* (full width at half maximum) and *θ* (Bragg angle) used in the crystallite size calculation based on the Scherrer equation are given in the ESI as Table S1.†

**Table 1:** Crystallographic parameters of the produced PAM/NiO NC

Sample	Crystallite size, (nm)	Crystallinity degree	Dislocation density *δ*	Microstrain, *ε*
PAM/NiO NC	26.33	0.125	0.001	−0.0065

### Crystallite characteristics determination using several models

#### Monshi–Scherrer method (Modified Scherrer equation)

The Monshi–Scherrer technique is another widely used model for determining crystallite size. In the fundamental classification of materials, two morphological categories exist: crystalline and amorphous forms. X-ray diffraction (XRD) patterns reveal that amorphous materials exhibit broader peaks, whereas crystalline structures display sharper peaks. The XRD method is applicable for determining the crystal size of crystalline structures only, as it cannot be used to calculate the crystal size of amorphous materials. If a given nanocrystal exhibits N distinct peaks within the angular range of *θ* from 0° to 90° or 2*θ* from 0° to 180°, each of these *N* peaks must correspond to the same crystallite size (*D*). However, this study highlights that the appropriate slope values chosen for the linear equation, which should be either greater or less than 1, remain uncertain.^56^ One advantage of the Modified Scherrer equation is its ability to minimize errors and utilize data from all or selected peaks, leading to a more accurate determination of the crystallite size (*D*).^57^ The Monshi–Scherrer equation is derived from the linear form of Scherrer's formula.^58^ We know the linear straight-line equation of Scherrer's equation is-6
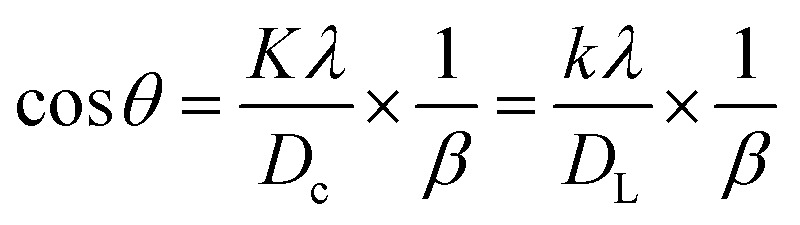
Here, *D*_L_ and *D*_c_ represents the crystalline size obtained using the linear straight line method.^59^

Rearranging the equation, we get7
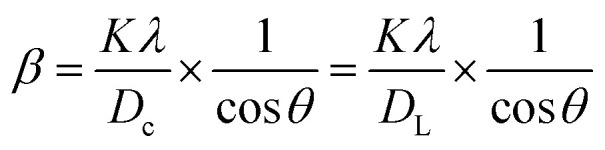


Now, taking ln on both sides of the equation, we can write-8
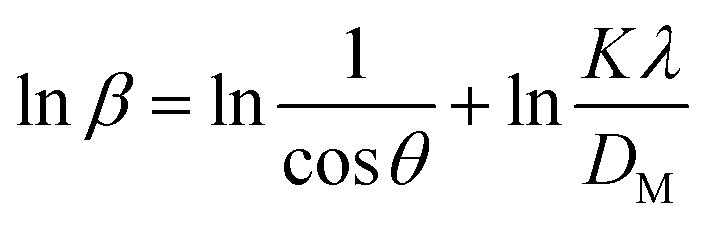


This formula indicates the equation for the Monshi–Scherrer method.

**Fig. 5:**
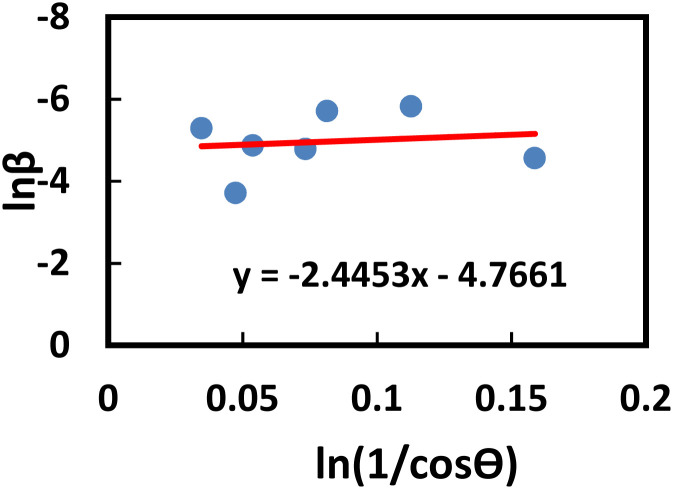
Determination of the crystallite size of the synthesized PAM/NiO NC by Monshi–Scherrer method.

If a straight-line plot is constructed with 
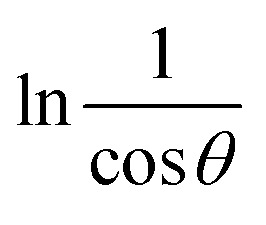
 on axis-*X* and ln *β* on axis-*Y*, as shown in Fig. 5, the intercept of the plot can be utilized to determine the crystallite size. Once the intercept is identified, its exponential value can be estimated:9
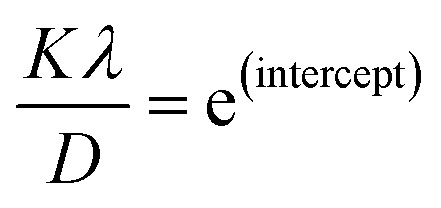


#### Williamson–Hall method (WHM)

The previous method focused exclusively on the impact of crystallite size on peak broadening in X-ray diffraction. However, it does not offer any insight into the lattice strain induced in the nanocrystal due to crystal imperfections.^60^ To address this issue, several advanced methods, including the Williamson–Hall method, have been developed. According to the Williamson–Hall approach, XRD peak broadening is influenced by both crystallite size and the presence of lattice strain and defects.^61^ So, the equation can be written as:

10 *β*_total_ = *β*_size_ + *β*_strain_

The WHM incorporates three models: the Uniform Deformation Model (UDM), the Uniform Deformation Energy Density Model (UDEDM), and the Uniform Stress Deformation Model (UDSM).

#### UDM

This model assumes that lattice strain is uniformly distributed along the crystallographic direction.^62^ According to the UDM, it is assumed that the crystal is isotropic in nature, and the material's properties are expected to be independent of the chosen direction of calculation.^63^ XRD peak broadening can be influenced by lattice strain, and the broadening resulting from strain can be expressed by the following equation-

11 *β*_strain_ = 4*ε* tan(*θ*)

If *β*_*hkl*_ represents the full width at half maximum intensity for various diffraction planes, the overall broadening of the XRD peak, attributed to both strain and crystallite size, can be described as:

12 *β*_*hkl*_ = *β*_size_ + *β*_strain_

where *β*_*hkl*_ denotes the width of the Bragg peak, and *β*_size_ and *β*_strain_ represent the broadening effects induced by size and strain, respectively. Substituting eqn (12) by (2) and (11), we get the relationship-13
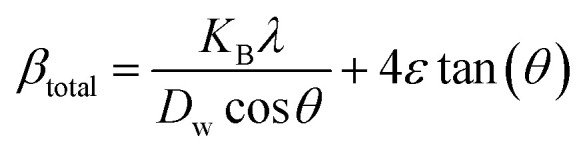


Rearranging eqn (13), we get,14
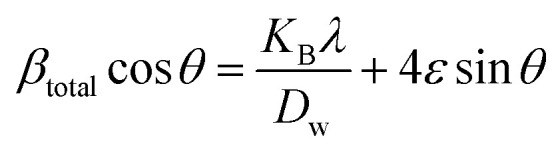


**Fig. 6:**
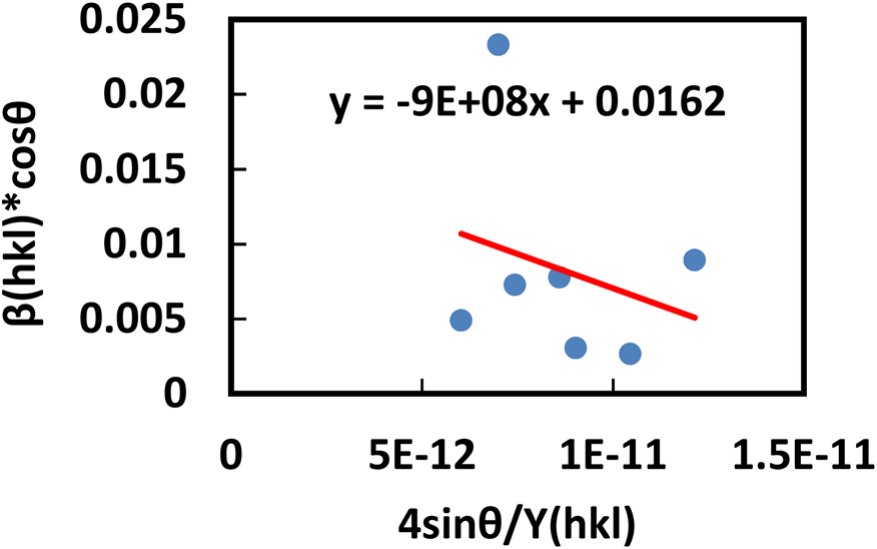
Determination of the crystallite size of the synthesized PAM/NiO NC by UDM.

This equation corresponds to the UDM, where *D*_w_ and *ε* represent the size of the crystal and microstrain values, respectively. The calculation of size and lattice strain can be easily performed using a graph that plots *β*_total_ cos *θ* against 4 sin *θ*. In Fig. 6, *D*_w_ can be obtained, and the slope of the graph allows for the determination of *ε*.

#### USDM

According to the UDM, the crystal is presumed to be isotropic and homogeneous. However, this assumption does not hold true in many cases. To better represent the actual conditions, an anisotropic approach was developed.^64^ USDM is a modified version of the WHM, incorporating anisotropic strain. This model assumes that deformation stress is uniformly distributed across all lattice planes and that the microstrain is minimal. It is particularly applicable when the strain in the nanocrystal is very small. Hooke's law defines the relationship between stress and strain, stating that, within the elastic limit, stress and strain are linearly proportional. However, once the material exceeds its elastic limit and strain increases, the uniform stress deviates from this linear relationship.^65,66^ Mathematically, that is-

15 *σ* = *εY*_*hkl*_

Here, *σ* represents the uniform stress of the crystal, *Y*_*hkl*_ is Young's modulus (modulus of elasticity), and *ε* denotes strain. By substituting the strain term from eqn (14) with (15), a new relationship can be derived.16
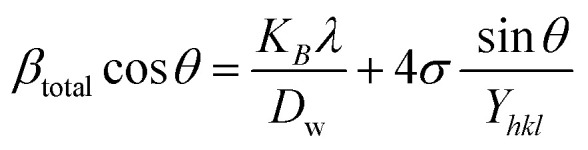


**Fig. 7:**
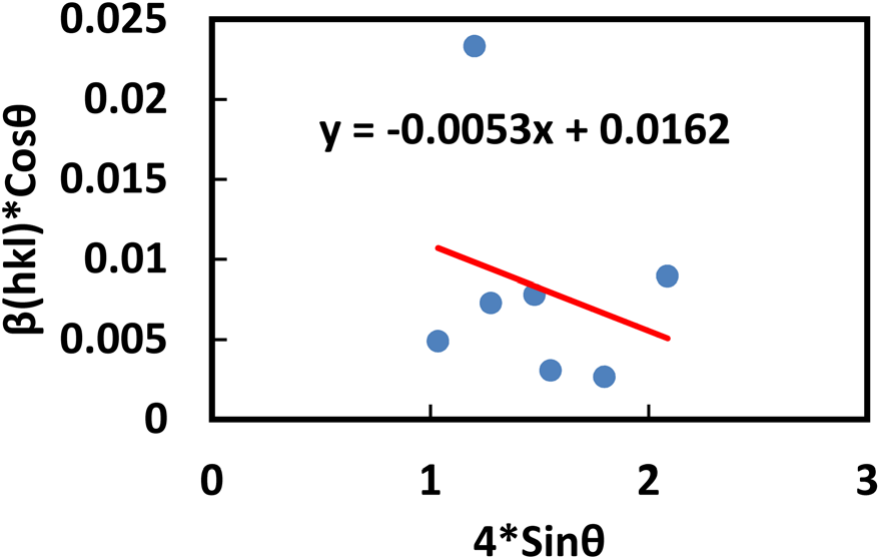
Determination of the crystallite size of the synthesized PAM/NiO NC by USDM.

This is the modified equation of the WHM, accounting for uniform stress across all crystallographic directions. By plotting a graph of 
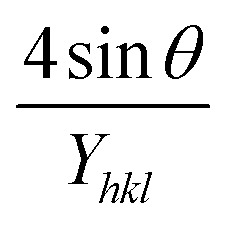
 against *β*_total_ cos *θ* in Fig. 7, the crystallite size and uniform deformation stress can be determined from the *Y*-intercept and slope, respectively.

#### UDEDM

As stated in the UDM, the crystal is presumed to be homogeneous and isotropic. However, many crystals are actually anisotropic due to lattice deformation. To address this, the USDM was developed. Furthermore, the stress-strain relationship is no longer linear, as demonstrated in the USDM, when the crystal's energy density (*u*) is considered.^67^ Crystal imperfections arise from various defects, agglomerates, and dislocations. Therefore, Williamson–Hall proposed an alternative method in which energy density is treated as a function of material strain. This model is suitable for calculating the values of *D*, *ε*, *σ*, and *u* (energy per unit volume).^68^ For an elastic system that follows Hooke's law, the energy density or energy per unit volume can be derived from the following expression-17
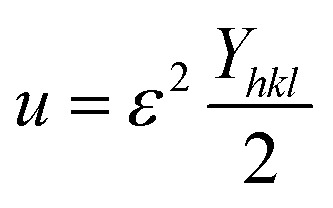


By rearranging the equation, it can be written as,
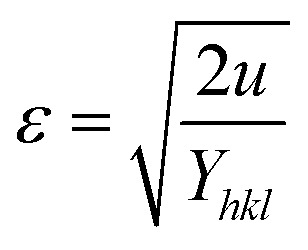


So, eqn (14) can be rewritten based on energy density and strain relationship -18
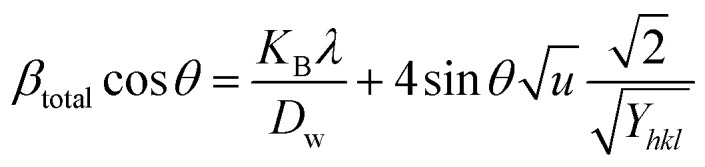


**Fig. 8:**
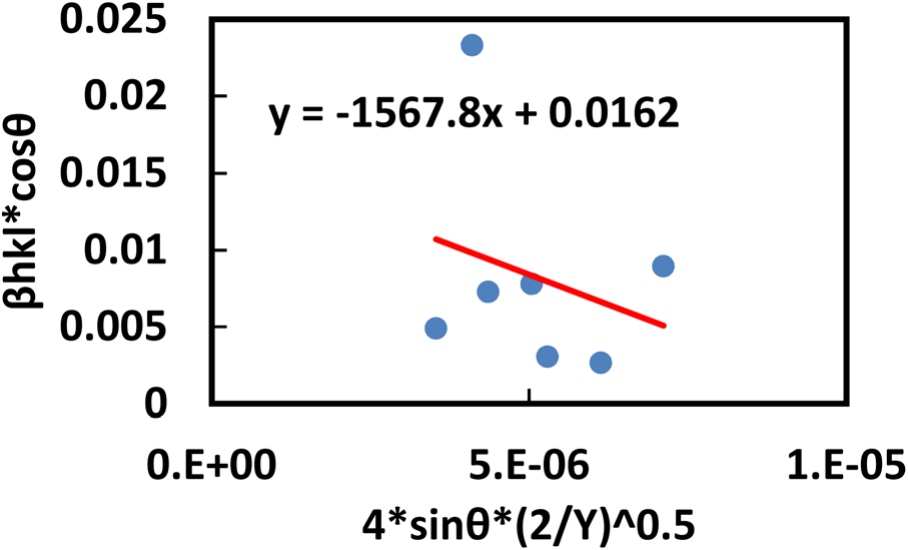
Determination of the crystallite size of the synthesized PAM/NiO NC by UDEDM.

The uniform energy density and crystallite size can be easily calculated from the straight-line curve in Fig. 8, which plots *β*_total_ cos *θ vs.*
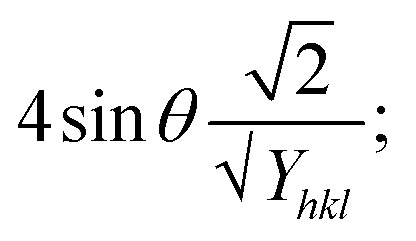
 The *y*-intercept and slope are used to determine the uniform energy density and the crystallite size, respectively. Additionally, the microstrain of the lattice can be estimated using the value of *Y*_*hkl*_.

#### Size-strain plot (SSP)

The line broadening appears to be isotropic, as indicated by the WHM.^69^ This implies that microstrain contributed to preserving the isotropy of the diffraction domains. However, by analyzing an average SSP, a more accurate assessment of the size–strain parameters can be obtained in the case of isotropic line broadening. One benefit of this method is that it provides data from high-angle measurements, where accuracy is typically lower, but with reduced weight.^70^

This method relies on the Gaussian and Lorentz functions, with the strain profile described by the Gaussian function and the crystallite size characterized by the Lorentz function.^71,72^ Therefore, the relationship between the Gaussian and Lorentz functions and the total broadening can be expressed as:

19 *β*_total_ = *β*_L_ + *β*_G_

Here, *β*_L_and *β*_G_ represent the peak broadening caused by the Lorentz and Gaussian functions, respectively.

SSP also has the advantage of giving more weight to data obtained from measurements at low angles, where accuracy is generally higher.^61^ Thus, the equation for estimating SSP is given by the following expression-20
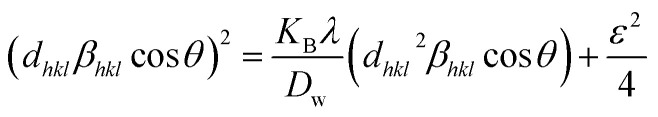
In this context, *ε* denotes the apparent strain, *K* is a shape-dependent constant (with *K* = 0.94 for spherical particles), and *d*_(*hkl*)_ is the lattice spacing between the (*hkl*) planes.

**Fig. 9:**
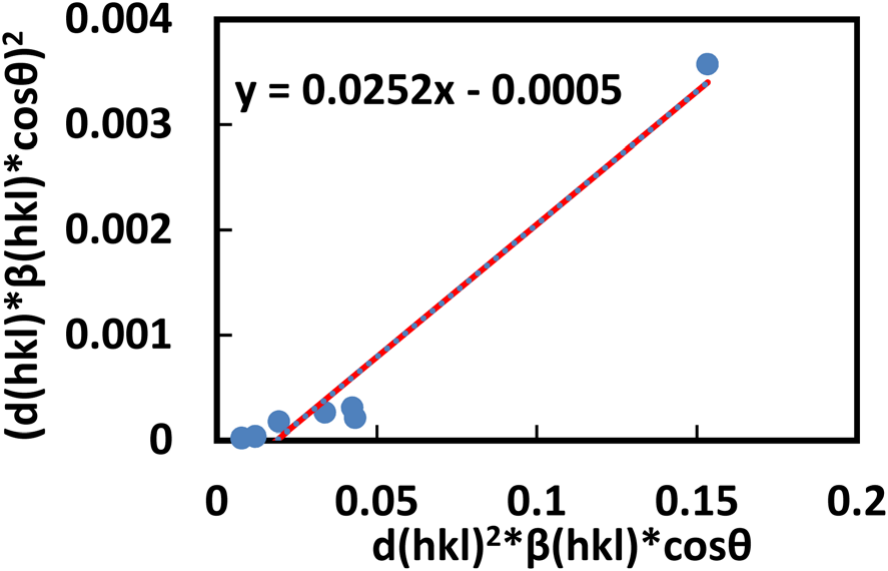
Determination of the crystallite size of the synthesized PAM/NiO NC by SSP.

The crystallite size and strain can be obtained by plotting a graph (d*hkl*^2^β*hkl* cos θ) *vs.* (*d*_hkl_*β*_hkl_cos*θ*)^2^in Fig. 9. The strain can be estimated by calculating the square root of the *y*-intercept, and the size of the crystallite can be estimated from the slope.

#### Halder–Wagner Method

In the earlier SSP method, it is assumed that the overall XRD peak broadening is governed by the Gaussian and Lorentz functions. However, in practical XRD analysis, the peak broadening of crystallites does not strictly follow either the Lorentz or Gaussian functions.^57^ The Gaussian function aligns well with the XRD peak but does not accurately match its tails. Conversely, the Lorentz function fits the tails of the XRD peak perfectly but fails to match the peak itself. To address this issue, the Halder–Wagner method was developed. This method assumes that the peak broadening follows a symmetric Voigt function, which forms the foundation of its core approach.^73^ According to this method, the physical profiles of the full width at half maximum (FWHM) for the Voigt function can be considered as:

21 *β*_*hkl*_^2^ = *β*_L_*β*_*hkl*_ + *β*_G_^2^

In this equation, the full-width half maxima caused by the Lorentz and Gaussian functions are denoted by *β*_L_ and *β*_G_.

Additionally, this method offers advantages as it focuses on peaks at low and medium angles, where diffraction peak overlap is minimal.^61^ Therefore, the relationship between lattice strain and crystallite size can be expressed as:22
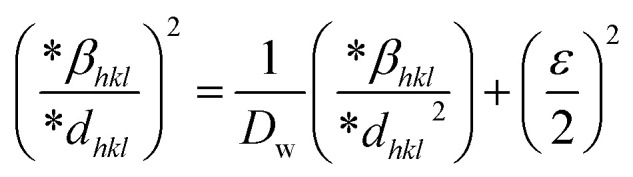
where *β_*hkl*_ = *β*_*hkl*_ cos(*θ*)/*λ*. *d*_*hkl*_ = 2*d*_*hkl*_ sin(*θ*)/*λ*. By plotting 
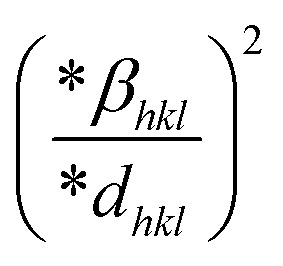
 across *Y*-axis and 
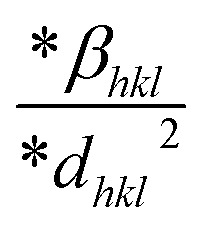
 term across the *X*-axis, the value of crystal strain and crystallite size can be measured.

**Fig. 10:**
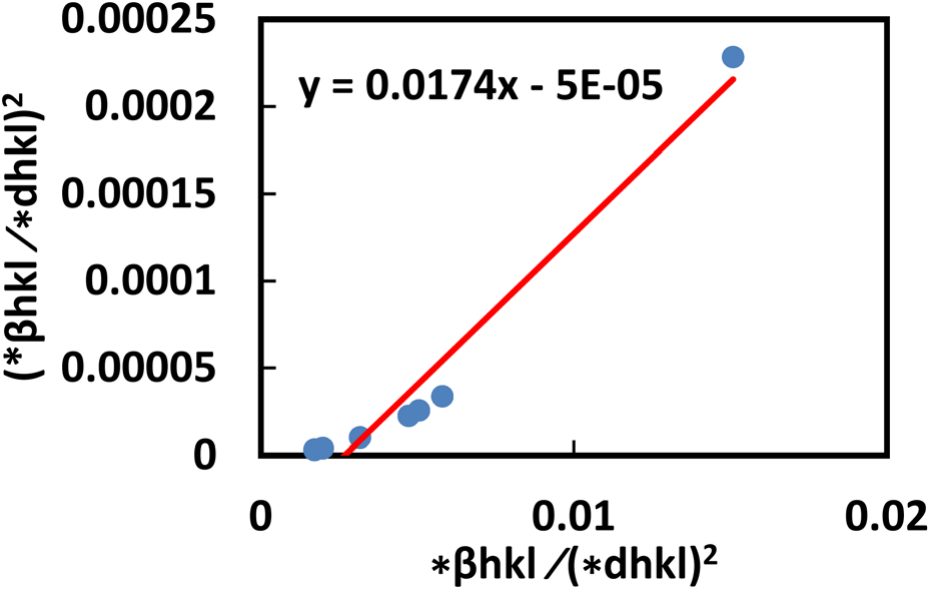
Determination of the crystallite size of the synthesized PAM/NiO NC by Halder -Wagner Method.

The *y*-intercept of the straight line in Fig. 10 is used to calculate the crystallite size, while the slope of the line is employed to determine the inherent strain of the nanocrystal.

#### Sahadat–Scherrer model

Crystalline materials with defined crystallite sizes are crucial for the effective use of nanocrystals. Several methods, including the Scherrer method, have been discussed earlier to calculate the intrinsic strain and crystallite size of nanocrystals. Recently, the Sahadat-Scherrer model was modified to address the limitations of previous models, enabling a more accurate determination of crystallite size.^74^ The strain-induced broadening was deemed negligible, and the model was based on removing instrumental broadening effects from the overall peak broadening. It was assumed that the peak widening was solely due to crystallite size, with the average crystallite size calculated using the derived equation, which is based on the original formulation.^57^ A straight line was plotted using the origin to calculate the crystallite size by considering all reflections in this model equation. The crystallite size was determined from the slope of the equation, which was compared to the standard linear equation (*y* = *mx*).^75^ This model's specifications are available in the literature.^76^ The relation is,23
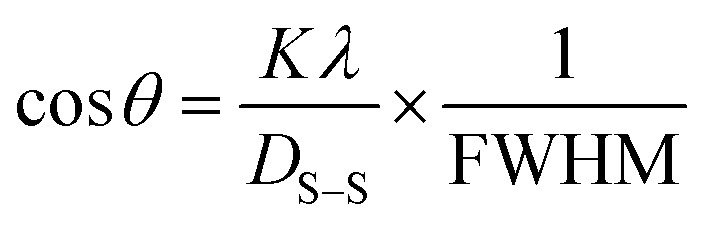


**Fig. 11:**
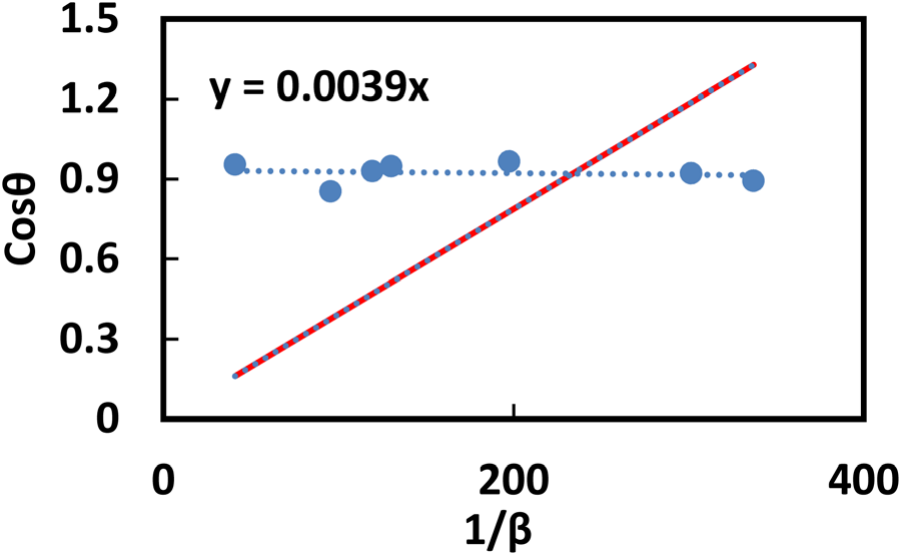
Determination of the synthesized PAM/NiO NC by Sahadat–Scherrer model.

This is the mathematical equation for the Shahadat–Scherrer model. One advantage of this method is that it allows for a straightforward calculation of crystallite size (*D*_s-s_).^77^ By plotting the graph from the result of cos *θ* and 
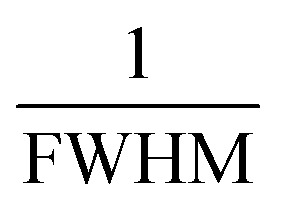
 on *x* and *y*-axis respectively in Fig. 11, we get a straight line. The slope of the straight line gives us the value of crystallite size. The other straight line is utilized for the evaluation of crystalline size by taking the slope 
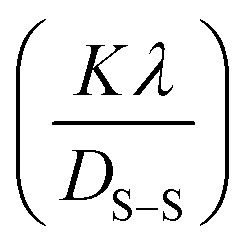
 of eqn (23). As the second straight line does not intersect the *y*-axis, the result will be more accurate.^78^Table 2 represents various crystallographic findings of synthesized PAM/NiO NC by the XRD models discussed above.

**Table 2:** Crystallographic findings of synthesized PAM/NiO NC

	Model name	Crystallite size, *D* (nm); strain, *ε* (N m^−2^); energy density, *u* (J m^−3^); stress, *σ*
		PAM/NiO NC
Monshi–Scherrer model		*D* = 16.29 nm
Williamson–Hall model	UDM	*D* = 8.55 nm
		*ε* = 0.0053
	USDM	*D* = 8.55 nm
		*σ* = 9 × 10^8^
	UDEDM	*D* = 8.55 nm
		*u* = 2.4 × 10^6^
Size-strain plot		*D* = 5.5 nm
		*ε* = 0.04
Halder–Wagner model		*D* = 5.7 nm
		*ε* = 0.003
Sahadat–Scherrer model		*D* = 35.01 nm

### Thermo-gravimetric analysis (TGA)

**Fig. 12:**
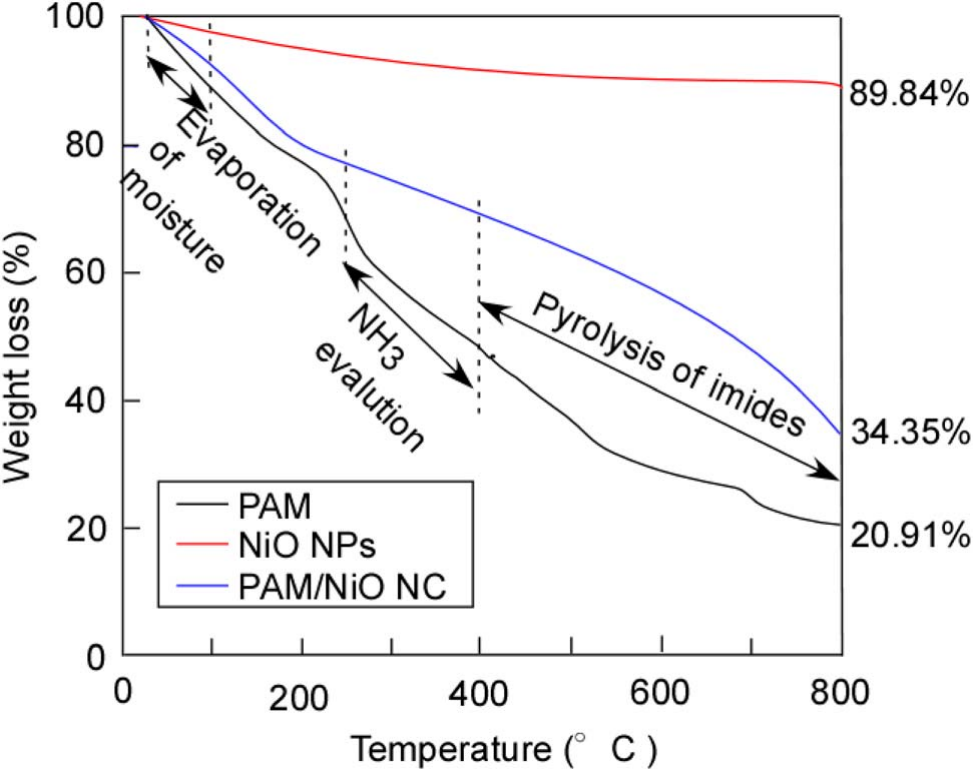
The TGA thermogram of (−) PAM, (−) NPs, (−) NC.

TGA is performed by gradually increasing the temperature and recording the weight loss as a function of temperature. This technique measures changes in the material's weight under a controlled atmospheric condition to assess its thermal stability. Fig. 12 represents the TG curve of NiO NPs, PAM, and PAM/NiO NC. For NiO NPs, the weight loss was only 10.16%, this could be attributed to the dehydration of lattice hydroxyl groups that are strongly bonded to the NiO, as indicated by the O–H bands in the FT-IR spectrum. This indicates that the synthesized NPs are pure. Not much foreign materials or organic substances were present in the NiO NPs, which suggests the sample's thermal stability. The TG curves of PAM and PAM/NiO NC show three stages of weight loss. The first weight loss below 200 °C can be attributed to the evaporation of physically or chemically adsorbed moisture in the polymer and nanocomposite surface.^20,34^ The second step of weight loss between 250–400 °C was due to NH_3_ evolution due to the imidization reaction between the amide groups of the monomer units.^20^ In this step, the weight loss for PAM and PAM/NiO NC were about 50% and 30% respectively. The weight loss observed in the third step above 400 °C was attributed by the pyrolysis of imides generated in the second step. A noticeable difference is observed between PAM and PAM/NiO NC in the 400–800 °C temperature range. PAM, being an organic polymer, burns completely at lower temperatures, resulting in a maximum weight loss of 20.91%.^79^ In contrast, PAM/NiO NC is a composite material where the polymer PAM is modified by embedding NiO nanoparticles (NPs). A higher weight loss of 34.35% was observed for PAM/NiO NC, which is attributed to the decomposition of the organic polymer embedded within the NiO NPs.^80^ This indicates that the incorporation of NiO NPs enhances the thermal stability of PAM.

### Scanning electron microscope (SEM) analysis

**Fig. 13:**
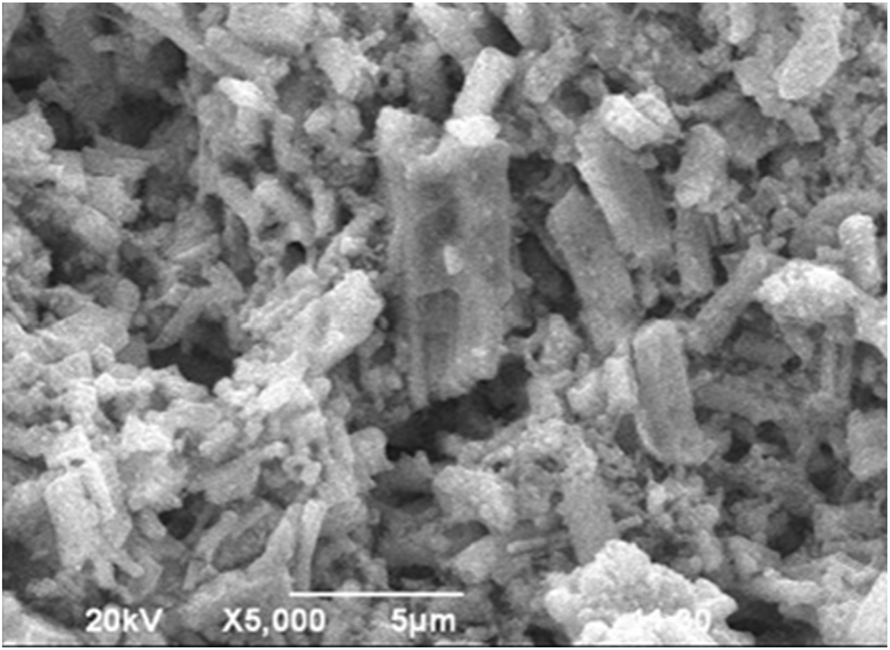
SEM image of PAM/NiO NC at ×5000 magnification.

The SEM micrograph of the synthesized PAM/NiO NC at ×5000 magnification (Fig. 13) confirms the formation of a hybrid nanocomposite. Literature shows that pure PAM polymer exhibits a smooth sheet-like morphology, whereas the PAM/NiO NC displays a rough surface with agglomeration due to the distribution of NiO NPs in the polymer matrix. NiO NPs are incorporated both on the surface and within the PAM matrix. The concurrent synthesis of NiO NPs and polymerization of the AM monomer guarantees a uniform dispersion of the NPs within the matrix. Similar morphological patterns have been observed in previous studies.^81,82^ As polyacrylamide acts as both a dispersing agent and a coupling agent,^83^ SEM shows an apparent size several times higher than XRD data due to particle aggregation in the stable sol stage, where polyacrylamide facilitates nanoparticle deposition. This aggregation leads to larger structures differing from XRD data. The incorporation of nanosized particles enhances the active surface area of the polymer matrix, imparting properties distinct from those of the pure polymer.

### Antimicrobial activity

**Fig. 14:**
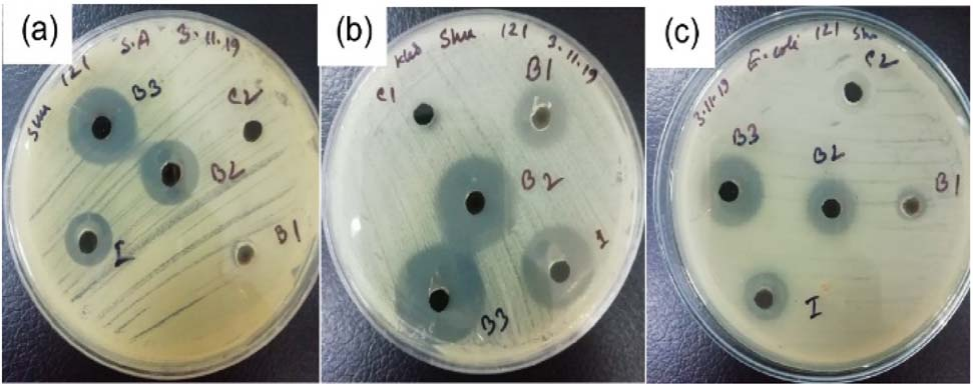
Antibacterial study to determine the of inhibition zone by varying concentrations (*B*_1_, *B*_2_, *B*_3_) of PAM/NiO NC against (a) *S. aureus*, (b) *Klebsiella* spp. and (c) *E. coli* bacterial strains.

The *in vitro* antibacterial activity of PAM/NiO NC suspensions at varying concentrations was evaluated against Gram-positive (*Staphylococcus aureus*) and Gram-negative (*Klebsiella* spp., and *E. coli*) bacteria. The inhibition zones around PAM/NiO NC samples at different concentrations are presented in Fig. 14.

**Fig. 15:**
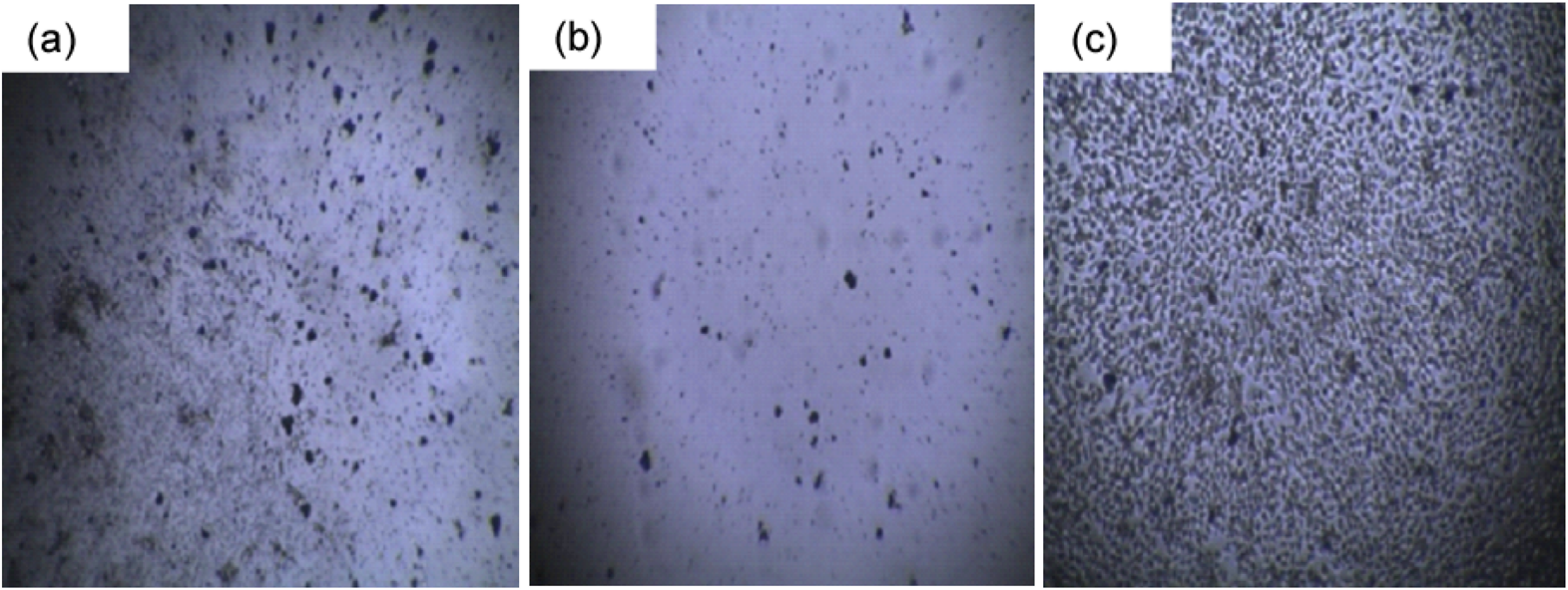
Images of the cytotoxic effect of PAM/NiO NC on (a) VERO; (b) HELA; (c) BHK-21 cell line.

The inhibitory activity was measured on the diameter of the inhibition zone. The contact area was used to evaluate growth inhibition underneath. From Fig. 15 and Table 3, it is visible that the nanocomposites exhibited excellent antibacterial activity against all G-negative bacterial strains, particularly *Klebsiella* spp. and *E. coli* irrespective of concentrations. The PAM/NiO NC displayed a great broad-spectrum antimicrobial activity with inhibition zones ranging from 7–27 mm against all human pathogenic bacteria. Synthesized NC showed the strongest antibacterial activity at its highest concentration and the weakest activity at the lowest concentration against all pathogenic bacteria. The inhibition zone was observed to expand with increasing NC concentration. Moreover, the PAM/NiO NC demonstrated a higher antibacterial activity for the G-negative (23–27 mm) than for the G-positive bacteria (16–19 mm), at a concentration ≥20 g L^−1^ which is in agreement with other findings.^42,48^ The most sensitive bacterial strain to PAM/NiO NC was *Klebsiella* spp., which showed the largest inhibition zone (27 mm at 30 g L^−1^) among all tested organisms. The strong antibacterial response against *Klebsiella* spp. indicates that PAM/NiO NC holds promise as an effective antibacterial agent, especially for Gram-negative pathogens. As pure PAM shows almost no antibacterial property,^51^ these findings clearly imply that the antibacterial activity of the synthesized nanocomposite is basically due to the NiO NPs that were impregnated into the PAM matrix. The antibacterial potential observed can be attributed to several factors, including particle size and shape, specific surface area, and material purity. While the precise mechanism underlying the antibacterial activity of metal oxide nanomaterials is not fully understood, several factors contribute to this activity. The generation of reactive oxygen species (ROS) by nanomaterials is considered a primary factor responsible for their antibacterial properties.^36,46^ This process causes significant damage to lipids, proteins, amino acids, DNA, and mitochondria, ultimately leading to cell death.^36,84^

**Table 3:** The inhibition zone diameter of PAM/NiO NC at various concentrations was measured against G-positive and G-negative bacteria^a^

Organisms	Diameter of inhibition zone		
	5 g per L conc. (B1)	20 g per L conc. (B2)	30 g per L conc. (B3)
*S. aureus* (Gram positive)	*R*	16 mm	19 mm
*Klebsiella* spp. (Gram negative)	14 mm	23 mm	27 mm
*E. coli* (Gram negative)	7 mm	14 mm	23 mm

a R = resistant to nanocomposites.

The NiO NPs in PAM/NiO NC have the capability to penetrate the cell membrane, disrupting the intracellular metabolism of Ca(ii) ions and subsequently causing cellular damage.^36^ It can also be stated that NiO NPs possess the cabability to interact with the functional groups in proteins, leading to the denaturation and, subsequently, cell death.^47^ Cell damage of bacteria is further induced by the interaction of NiO NPs with phosphorus- and sulfur-containing compounds, such as DNA, and by the deactivation of bacterial enzymes.^84^ Another cause of the antibacterial activity of PAM/NiO NC could be the dissociation of Ni^2+^ ions from the NiO NPs of NCs.^42,48,84^ Released Ni^2+^ ions can easily penetrate the cell wall, which later disturbs the electron transport system, and oxidative stress gets induced, resulting in cell damage to bacteria.^48^ A comparative study of antibacterial activities of NiO nanoparticles and PAM polymer and NiO nanoparticles-based nanocomposite synthesized by various methods given in Table 4 indicates that PAM and NiO NPs based several nanocomposites exhibited moderate to strong antibacterial activity against both G-positive and G-negative bacterial strains in all cases. Besides, pure NiO NPs showed moderate activity in reports of others (Table 4), which triggered the expectation that PAM/NiO NC should also possess good antibacterial activity if synthesized. In this study, our synthesized PAM/NiO NC, which was not studied before, showed strong antibacterial activity against all types of pathogenic bacteria, as was estimated by analyzing previous works of others.^25,48,84–88^

**Table 4:** A comparison of the antibacterial activity observed in this study with that reported in previous works

Synthesis method	Materials	Concentration (g L^−1^)	Bacterial strains	Level of activity	References
*In situ* emulsion polymerization	PAM/ZnO NCs	1	Gram positive	Moderate	84
			Gram negative	Moderate	
			Gram positive	Strong	
Sol–gel method	PAM/Ce(iv) silicophosphate NCs	0.32	Gram negative	Strong	85
Sol–gel method	PAM/Zr(iv) vanadophosphate NCs	0.4	Gram positive	Strong	86
			Gram negative	Strong	
			Gram positive	Moderate	
		0.35			
Chemical	PVA/NiO NCs	0.02	Gram negative	Weak	87
Chemical	PVA -MF/NiO NCs	N/A	Gram positive	Strong	89
			Gram negative	Strong	
Green synthesis	NiO NPs	0.6	Gram positive	Weak	48
			Gram negative	Weak	
Microwave assisted	NiO NPs	0.04	Gram positive	Moderate	25
			Gram negative	Moderate	
Microwave assisted	PAM/NiO NCs	20	Gram positive	Strong	This study
			Gram negative	Very strong	

### Cytotoxicity analysis

In this research, the cytotoxicity of PAM/NiO NC on VERO (a kidney epithelial cell line derived from an African green monkey), HELA (a human cervical cancer cell line), and BHK-21 (a fibroblast cell line from baby hamster kidney) were investigated. VERO cells are widely used in toxicology and virology studies due to their sensitivity and stable growth characteristics.^90^ Including VERO cells in the cytotoxicity assessment of PAM/NiO nanocomposites allows for evaluating their biocompatibility with normal, non-cancerous mammalian cells, thereby providing insight into potential toxicity effects in healthy tissues. On the other hand, the inclusion of HELA cells is essential for evaluating the potential anticancer properties or selective cytotoxicity of the nanocomposite toward malignant cells.^91^ Similarly, BHK-21 cells, being non-cancerous fibroblasts, serve as a representative model for normal mammalian cells, allowing assessment of the biocompatibility and safety of PAM/NiO NC for healthy tissues.^92^ The use of both cancerous and non-cancerous cell types enables a comprehensive understanding of the nanocomposite's differential cytotoxic effects, which is crucial for determining its suitability for biomedical applications such as cancer therapy or antimicrobial coatings.

**Fig. 16:**
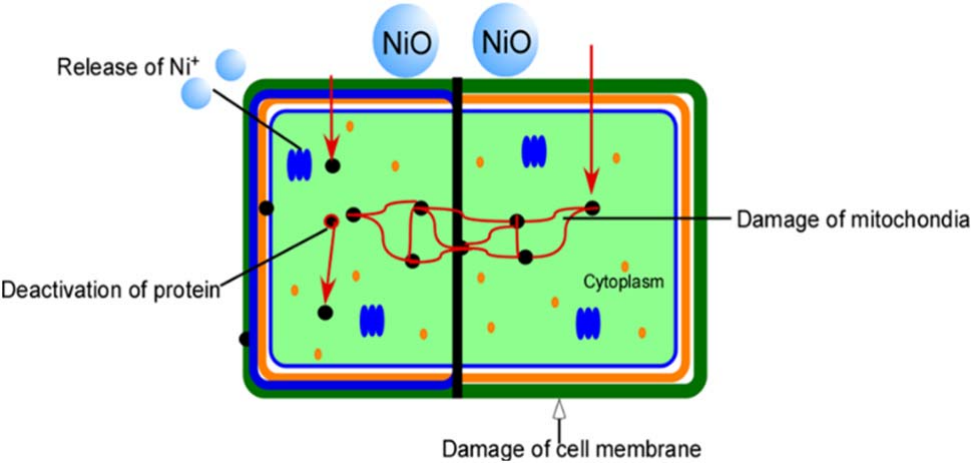
Schematic presentation of NiO NPs induced cytotoxicity.

From the results (Fig. 15 and Table 5), it is evident that the toxicity of PAM/NiO NC against HELA, a human cervical carcinoma cell, was maximum (less than 5% of cells survived). In contrast, it was not that harmful towards the BHK-21 cell line. Giving more research effort to this nanocomposite can be used for medical applications to kill malignant cancer cells. Like antibacterial performance, the cytotoxicity of PAM/NiO NC must also be attributed to the toxicity induced by NiO NPs. To understand the cytotoxic effects and the possible mechanism of action of NiO NPs on various tissues and cells, investigations reported by others have been considered.^36,93,94^ Studies suggested that excessive production of reactive oxygen species (ROS) and oxidative stress could be one of the possible mechanisms of nanoparticle toxicity.^36,93^ ROS generally induce DNA damage, including various oxidized base lesions, abasic sites, and single and double-strand breaks. These damages can be cytotoxic, genotoxic, or mutagenic.^93^ We found our synthesized NC to be apoptosis towards HELA cell which is in agreement with the finding that NP-induced oxidative stress leads to DNA damage and apoptosis.^36,93^ The oxidative modification of proteins and lipids by reactive oxygen species generates toxic electrophilic species, such as hydroxyl radicals (–OH), superoxide anions (O_2_^−^), and hydrogen peroxide (H_2_O_2_), which disrupt cellular pathways and interfere with signal transduction, leading to toxicity. Moreover, metal oxides interact with proteins and salts, which are internalized by the cell membrane through endocytosis, thereby impacting cellular metabolism *via* the uptake of these external substances.^36^Fig. 16 presents a schematic illustration of the potential effects of toxicity induced by the NiO NPs in PAM/NiO NC.

**Table 5:** Survival of cells after treatment with PAM-NiO NC

Sample	Dose	Survival of cells		
		VERO	HELA	BHK-21
PAM/NiO NC	30 mg mL^−1^	10–20%	<5%	>95%

### Photocatalytic activity

**Fig. 17:**
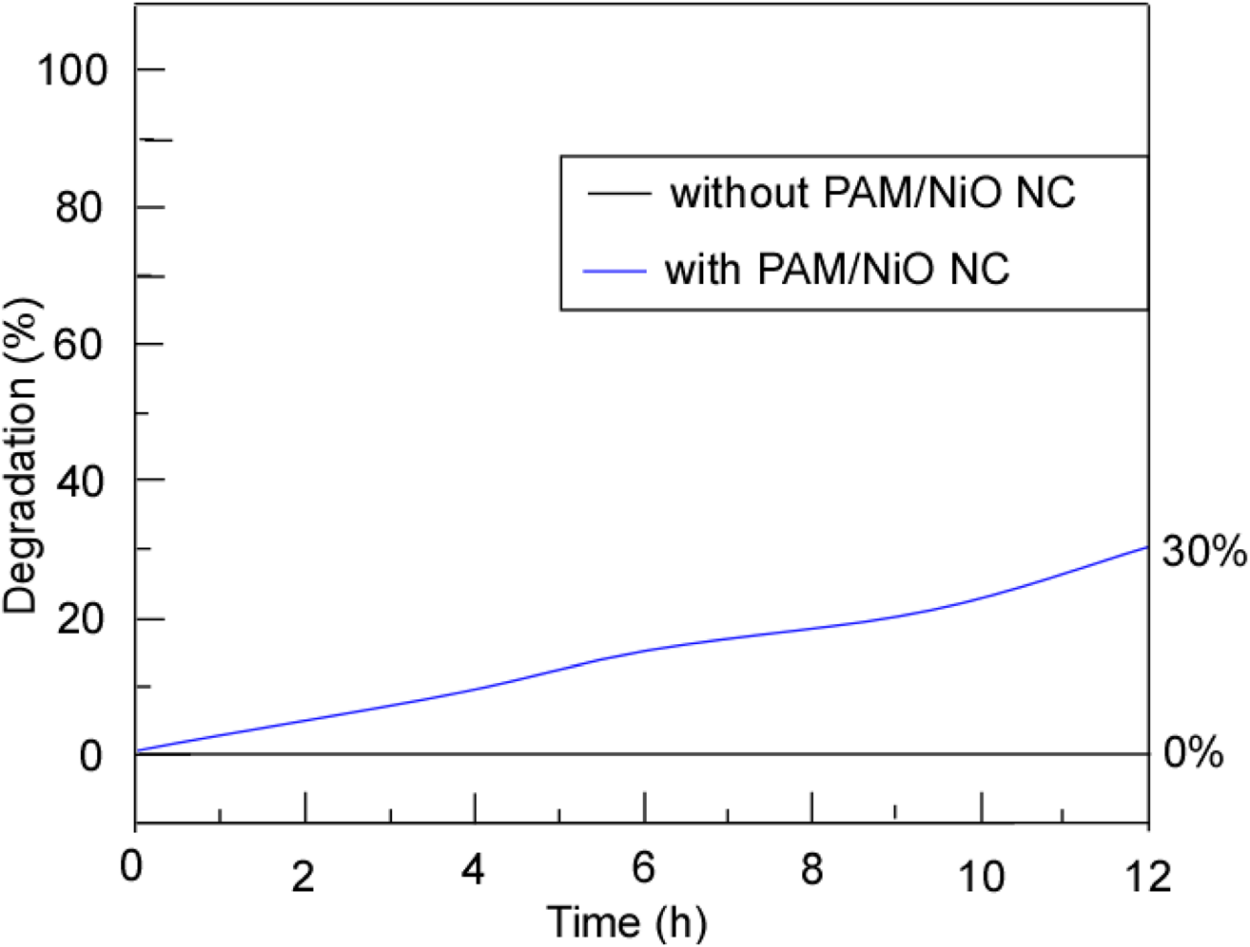
Photocatalytic degradation of Remazol yellow RR dye with (−) and without (−) PAM/NiO NC in the presence of sunlight.

The photocatalytic degradation of Remazol Yellow RR dye using PAM/NiO NC was examined at a catalyst concentration of 0.2 g L^−1^ under sunlight irradiation, with the corresponding results presented in Fig. 17. The photocatalytic efficiency of PAM/NiO NC was evaluated by tracking the changes in the absorption maxima of Remazol Yellow RR at 420 nm. Photodegradation analysis was conducted for dye concentrations of 40 ppm.

Control experiments were performed to evaluate the extent of degradation in the absence of the photocatalyst, commonly referred to as photolysis. When the dye solution was irradiated with UV light without PAM/NiO NC (the photocatalyst), no degradation was identified. However, in the presence of the nanocomposite, photodegradation occurred. However, in spite of sunlight exposure, PAM/NiO NC catalyst showed a very poor and confined rate of degradation (30% after 12 hours) for the Remazol yellow RR dye solution. Although a lot of reports suggested a very good photocatalytic activity by NiO and PAM-based nanocomposites,^85–88^ we found the opposite. Several studies have suggested that the catalyst's light absorption ability and the availability of active sites on its surface can notably impact the rate of photodegradation.^94^ At specific catalyst concentrations, agglomeration takes place, which hinders the ingress of UV light and causes diffraction. Consequently, only a limited number of NiO molecules are activated, resulting in insufficient hydroxyl radical formation for dye molecule degradation and, thus, reduced photodegradation efficiency.^36^ In this study, the limited photocatalytic activity may also be due to this agglomeration of NiO NPs of PAM/NiO NC.

## Conclusions

Microwave-assisted one-step synthesis of PAM/NiO NC has been demonstrated as a fast, uniform, energy-efficient, and environment-friendly approach. The composites' crystallite sizes were found to fall into the nano-range (1–100 nm) using the Scherrer equation and several different models. All the characterization confirms that a PAM/NiO NC has been successfully synthesized. The antibacterial, cytotoxicity, and photodegradation properties of the prepared PAM/NiO NC were assessed. Notable cytotoxic effects were observed against HELA cell lines, along with enhanced antibacterial activity against both Gram-positive (*S. aureus-8a*) and Gram-negative (*Klebsiella* spp.*SK4, Escherichia coli RN89*) bacteria. Comprehensive mechanisms underlying the aforementioned applications are also proposed and discussed. In conclusion, it can be stated that the microwave-assisted PAM/NiO NC is applicable in various biomedical and environmental sectors.

## Data availability

Data will be made available upon request.

## Author contributions

Mohammed Yusuf Miah conceived and designed the plan. Sukanta Halder synthesized and characterized the produced samples, analyzed the data, and wrote the draft and original manuscript. Shassatha Paul Saikat and Sanchita Dewanjee assisted in writing the draft. Md. Ashaduzzaman and Shukanta Bhowmik supervised the findings of this work. Shukanta Bhowmik supervised in writing and managed the required facilities.

## Conflicts of interest

There are no conflicts to declare.

## Supplementary Material

RA-015-D5RA02496J-s001
