# Supplementary material for: Microwave-assisted one-step synthesis of polyacrylamide/NiO nanocomposite for biomedical applications
Source: RSC Adv. 2025 Jun 5;15(24):18971–85. doi: 10.1039/d5ra02496j (PMC12138337; doi:10.1039/d5ra02496j)
Supplement: RA-015-D5RA02496J-s001 [file RA-015-D5RA02496J-s001.pdf]

Supplementary Information (SI) for RSC Advances.
This journal is © The Royal Society of Chemistry 2025

Supplementary information

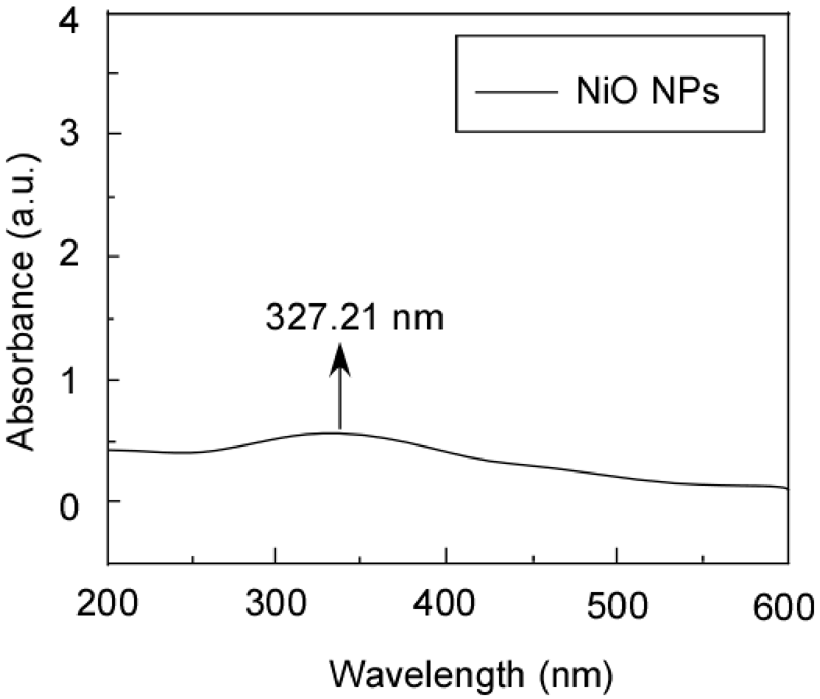

Figure S1: UV-Vis spectrum of NiO NPs

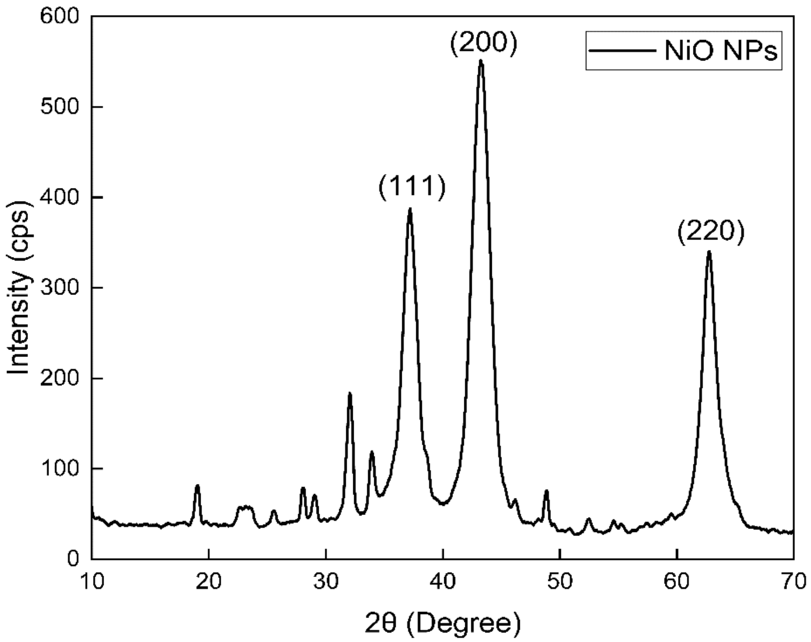

Figure S2: X-ray diffractogram of the NiO NPs

Table S1: The specific values of β (full width at half maximum) and θ (Bragg angle) used in the crystallite size calculation based on the Scherrer equation

θ (deg)	β (FWHM°)
15.0085	0.29
17.5	1.4
18.622	0.44
21.6825	0.48
22.815	0.19
26.6945	0.17
31.4325	0.6
